# Supplementary figures and images for: Parental Origin of Gsα Inactivation Differentially Affects Bone Remodeling in a Mouse Model of Albright Hereditary Osteodystrophy
Source: JBMR Plus. 2021 Nov 16;6(1):e10570. doi: 10.1002/jbm4.10570 (PMC8771002; doi:10.1002/jbm4.10570)

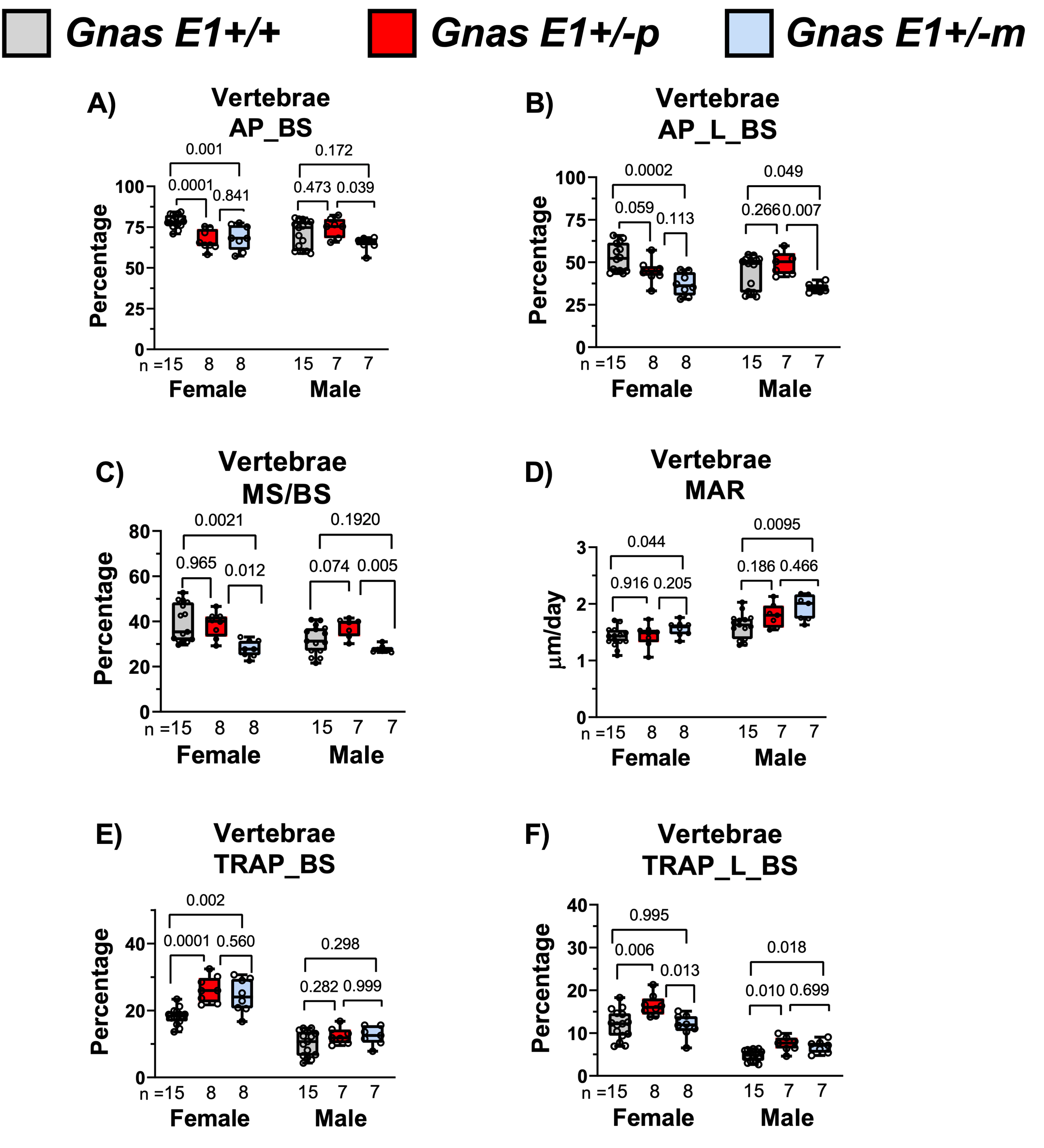

Supplement: Supplementary file 1 — Supplemental Fig. S1 Gnas heterozygous inactivation differentially affects bone formation and resorption in the lumbar vertebrae. (A) Significant reductions in total alkaline phosphatase activity on the bone surface (AP_BS) was observed within female Gnas E1+/−p and Gnas +/−m lumbar vertebrae sections compared to WT. No significant differences were observed in male specimens across all genotypes. (B) Gnas E1+/−m mice displayed a reduction in the total number of actively mineralizing osteoblasts (AP_L_BS) when compared to WT and Gnas E1+/−p mice. (C) Dynamic histomorphometry on the vertebral trabecular surface revealed Gnas E1+/−m mice displayed a significant reduction in MS/BS when compared to both WT and Gnas E1+/−p mice (D) Gnas E1+/−m mice displayed a significantly elevated mineral apposition rate on the vertebral trabecular surface when compared to WT. (E) Female Gnas E1+/−p mice demonstrated a significant increase in total TRAP activity on the bone surface (TRAP_BS) and (F) active bone remodeling sites (TRAP_L_BS) within the vertebrae when compared to WT mice. No significant variations were observed between female Gnas E1+/−m and WT mice. No significant phenotype was observed within male mice. Sample size per genotype per experiment is listed on each bar graph. All statistical tests completed using ANOVA with post‐hoc Tukey test for multiple comparisons, and p values are displayed for each comparison. [file JBM4-6-e10570-s001.tiff]

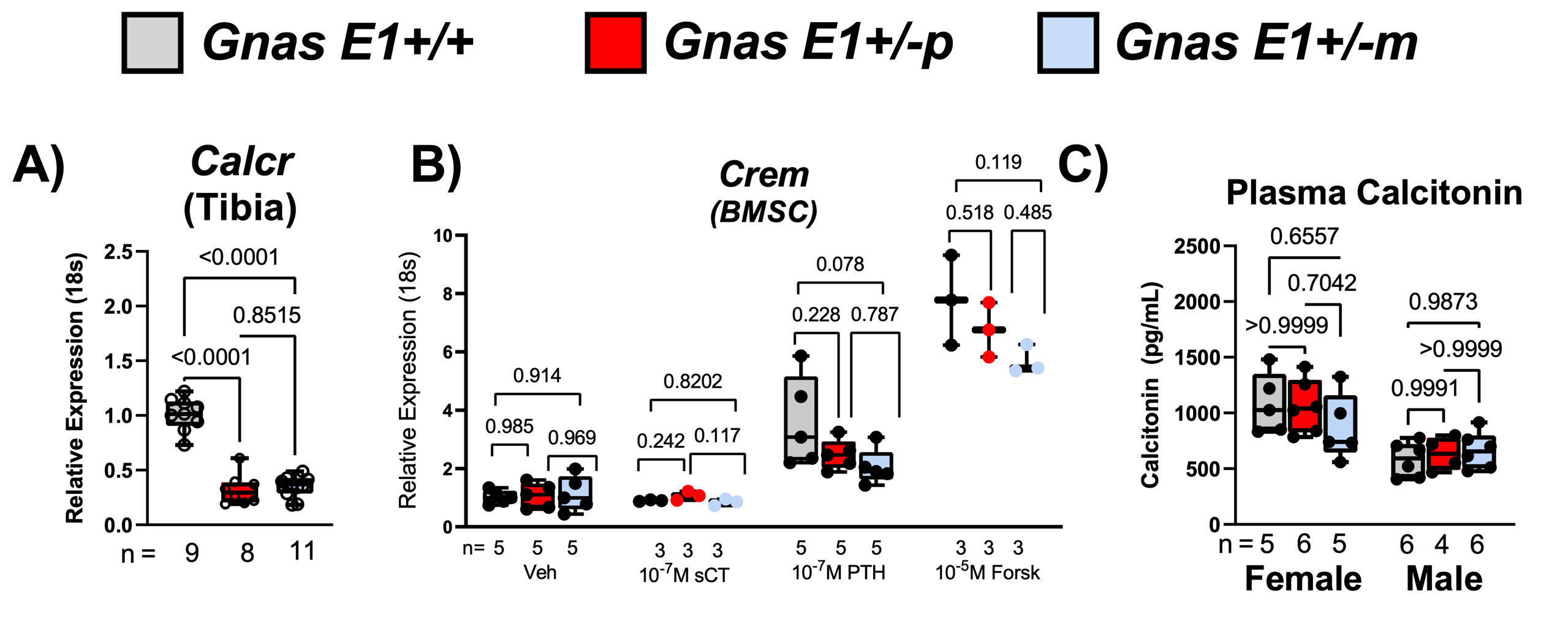

Supplement: Supplementary file 2 — Supplemental Fig. S2 (A) RT‐PCR analysis of flushed tibia diaphysis from male and female Gnas E1+/−p and Gnas E1+/−m mice displayed a reduced mRNA expression of Calcr when compared to WT. (B) RT‐PCR analysis of Crem mRNA expression of Gnas E1+/−m, Gnas E1+/−p, and WT bone marrow stromal cells (BMSCs) following exposure to salmon calcitonin (SCT), PTH, forskolin or vehicle controls for 6 hours. BMSCs overall displayed no significant response to sCT treatment. WT PTH‐treated BMSCs displayed a significant increase in Crem when compared to vehicle controls; however, Gnas E1+/−m and Gnas E1+/−p BMSCs displayed no significant changes compared to vehicle controls. No significant variations were observed between PTH treated WT and Gnas E1+/−m or Gnas E1+/−p BMSCs. (C) Plasma calcitonin measurements obtained from 12 week old WT and Gnas E1+/− mice revealed no significant variations in calcitonin levels. Sample size per genotype per experiment is listed on each bar graph. All statistical tests in (A) and (C) completed using ANOVA with post‐hoc Tukey test for multiple comparisons. Statistical analyses performed in (B) were completed using a two‐way ANOVA with post‐hoc Tukey test for multiple comparisons and p values are displayed for each comparison. [file JBM4-6-e10570-s002.tiff]
